# Perioperative Antibiotic Administration Review: An Analysis of Outpatient Surgeries at OhioHealth Doctors Hospital

**DOI:** 10.7759/cureus.95443

**Published:** 2025-10-26

**Authors:** Rayyan Bhutta, Thomas Shneker, Samantha Currier, Alexander Caskey, Vincent Relli-Dempsey, Matthias Franzen, Samir Patel

**Affiliations:** 1 Medical School, Ohio University Heritage College of Osteopathic Medicine, Dublin, USA; 2 Anesthesiology, OhioHealth Doctors Hospital, Columbus, USA; 3 Anesthesiology, Cleveland Clinic, Cleveland, USA

**Keywords:** anesthesiology practice, antibiotic administration, cefazolin, clinical practice guideline, surgical site infections (ssi), weight-based dosing

## Abstract

Introduction: A vital part of preventing surgical site infections (SSIs) is perioperative antibiotic prophylaxis. Antibiotics must be administered within a certain window of time, usually 15 to 60 minutes prior to the surgical incision, for them to be effective. The most frequently utilized medication, cefazolin, can be administered quickly by intravenous push (IVP), but other medications, such as vancomycin and clindamycin, need longer infusion times. Antibiotic efficacy declines, and the risk of SSIs is increased when scheduling requirements are not followed. With special focus on time, dosage, and variation by surgery type, this study evaluates adherence to perioperative antibiotic administration guidelines in outpatient surgeries at OhioHealth Doctors Hospital.

Methods: This retrospective study was conducted at OhioHealth Doctors Hospital in Columbus, Ohio. Patients aged 18 and older who underwent outpatient surgery between January 1, 2018, and December 31, 2021, were included. A total of 448 cases were analyzed. Data were extracted from electronic medical records and reviewed to assess antibiotic type, administration timing, dosing accuracy (based on patient weight), and surgery type. Descriptive statistics and appropriate statistical tests, including chi-square and confidence intervals, were used for analysis.

Results: Antibiotics were given to just 3.3% of patients over the advised pre-incision period. Antibiotics offered via IVP had a greater probability of being delivered on schedule (4.6%) compared to those that needed infusion pumps (0.7%, p = 0.0446). The most frequently administered antibiotic was cefazolin (65.6%), with 88.8% of doses being appropriately weight-adjusted. Nonetheless, there were notable differences in timing protocol adherence by surgical specialty, with numerous specialties displaying 0% compliance and orthopedic procedures exhibiting the highest adherence at 7.9%.

Conclusion: Adherence to perioperative timing standards was concerningly inadequate, even though antibiotic selection and dosage were generally suitable. The findings point to a substantial workflow and protocol enforcement gap at the system level. Operational adjustments like real-time warnings, checklists integrated with electronic health records (EHRs), and simpler delivery techniques may increase adherence and reduce preventable SSIs. Future research is required to assess how these strategies influence patient outcomes.

## Introduction

Project lay summary

Patients undergoing outpatient surgery typically receive antibiotics to reduce the likelihood of developing a surgical site infection (SSI). To be most effective, antibiotic administration should begin before the surgery starts, with specific windows of administration based on the type of antibiotic used. Specifically, some antibiotics have a large therapeutic index and may be administered safely in a very short amount of time. By contrast, certain antibiotics require a longer duration of administration to prevent possible deleterious side effects, which often leads to the antibiotic not getting entirely administered prior to incision. Administration of antibiotics outside of the effective windows places patients at increased risk of developing an SSI. The purpose of this study was to determine whether patients undergoing outpatient surgeries at Doctors Hospital were receiving antibiotics prior to surgery, in alignment with evidence-based guidelines [1].

Background/rationale 

In 2018, community hospitals in the United States performed a total of 28 million surgical procedures, with outpatient surgeries reaching a historical all-time high of over 19.1 million surgeries [2]. These surgical procedures risk introducing patients to foreign microbes that are commonly found in the normal skin microflora, primarily staphylococcal and streptococcal species. SSIs are defined as an infection relating to an operative procedure on or adjacent to a surgical incision within 30 days of a procedure or within 90 days of implanted prosthetic material [3]. SSIs remain the leading cause of nosocomial infections in surgical patients, accounting for up to 300,000 infections annually [1]. SSIs represent a significant burden on the healthcare system by prolonging hospitalization, increasing readmissions, and reducing patient quality of life, with an associated mortality rate of 3% [4]. These infections are estimated to cost the healthcare system an excess of $3.3 billion annually [5]. Appropriate antimicrobial prophylaxis against SSIs remains a critical component of perioperative care to improve patient outcomes and curtail additional healthcare costs. 

Antibiotic prophylaxis for surgeries became widely accepted as standard of care in the 1970s. The first randomized, prospective clinical trial on the efficacy of antibiotic prophylaxis was in 1964 on abdominal operations [6]. After showing benefit, various other clinical trials went on to establish the evidence-based protocols for surgical procedures beyond operations involving the gastrointestinal tract. While not every surgical procedure has been extensively studied, the rationale for antimicrobial prophylaxis remains compelling for preventing SSIs. The recognition of well-known risk factors predisposing patients to acquiring an SSI plays an important role in perioperative care. Patient risk factors include female gender, older age, obesity, low serum albumin concentration, current infection, smoking, nutritional status, diabetes mellitus, and hyperglycemia regardless of diabetic status [7]. Anesthesiologist risk factors include the timing of initial antibiotic infusion, intraoperative redosing, glycemic control, avoidance of hypothermia, and goal-directed fluid therapy [2]. Other risk factors include procedure duration, instrument sterilization, and surgeon proficiency and technique.

In the majority of surgical procedures, cefazolin remains the agent of choice for antimicrobial prophylaxis. Cefazolin has been one of the most utilized antimicrobials as it protects against gram-positive bacteria typically found in skin flora. Cefazolin has a wide microbial coverage, relatively low cost, and a reliable safety profile, which allows it to be administered in three to five minutes [8]. This typically allows enough time for the medication to be administered after induction of general anesthesia, but more than 15 minutes before the start of the procedure. A previous study has suggested it takes anywhere from 20 to 60 minutes for cephalosporins to reach minimum inhibitory concentration (MIC) in target tissues [9]. Current antibiotic dosing guidelines may be weight-based or a standard dose, depending on the antibiotic. Cefazolin dosing is recommended at 2 grams (g) for adults weighing less than 120 kilograms (kg) and 3 g for those ≥120 kg [10]. When necessary, intraoperative redosing of cefazolin is recommended four hours after the initial preoperative dose, not from the beginning of the surgical incision. 

Penicillin is a commonly listed allergy on a patient’s chart [11]. Type 1 hypersensitivities are immunoglobulin E (IgE)-mediated and a consequence of mast and basophil cell degranulation due to prior IgE sensitization with previously contacted antigens, which may include penicillin. While type 1 hypersensitivity reactions between penicillins and cephalosporins are rare, current guidelines emphasize that cephalosporins should not be used in patients with a suspected or documented IgE-mediated penicillin allergy [10]. However, it is equally emphasized that cephalosporins can be safely used in patients with a documented penicillin allergy that is not a type 1 hypersensitivity reaction. This may include patients with documented penicillin reactions that include clarifiers that cephalosporins can be safely given or only experienced nonspecific symptoms such as nausea, vomiting, and diarrhea. True type 1 hypersensitivity reactions typically include hives, angioedema, wheezing, dyspnea, and/or anaphylaxis immediately or within one hour of administration. It is important to note that upwards of 80% of patients with IgE-mediated penicillin allergies lose their sensitivity after 10 years. Accurately identifying patients who are not truly sensitive to penicillin can decrease the need to switch from cefazolin to a different class of antibiotic.

Vancomycin is often the antibiotic chosen for gram-positive coverage when there is a contraindication to cefazolin, most commonly in cases of IgE-mediated allergies. Current guidelines maintain the importance of initiating vancomycin infusion slowly to prevent histamine release and subsequent vancomycin infusion reaction (VIR), previously known as red man syndrome. While VIR is typically mild and presents as pruritus and a diffuse erythematous rash, more severe adverse effects include hypotension and potential cardiovascular collapse. Completing vancomycin infusion and achieving MIC within one hour of surgical incision has been associated with the best outcomes. Previously, vancomycin administration between 16 to 60 minutes before incision was associated with 11.6 times decreased odds of acquiring an SSI when compared to zero to 15 minutes [12]. Furthermore, completing vancomycin infusion before initial incision was found to have a significant decrease in mortality associated with SSIs (4.5% vs. 14.4%, p < 0.001) [13]. Redosing is generally not necessary due to vancomycin’s long half-life of four to six hours in patients with normal renal function. Notably, patients with chronic kidney disease (CKD) requiring vancomycin infusion require careful dosing monitoring due to the altered pharmacokinetics and high risk of nephrotoxicity [14,15]. 

Specific aims 

The purpose of this research study was to determine how well the Doctors Hospital Anesthesia Department adhered to clinical guidelines for perioperative antibiotic use in patients who underwent outpatient surgeries.

Aim 1 is to calculate the frequency with which antibiotics were administered at an appropriate time prior to surgery, defined as completion of antibiotic administration at least 15 minutes, but no more than 60 minutes before surgical incision, regardless of therapeutic index. We hypothesized that antibiotics that can be administered via an intravenous pump (IVP) (such as cephalosporins) were administered with the appropriate timing >95% of the time, but antibiotics that require an infusion pump (e.g., vancomycin, clindamycin, metronidazole, ciprofloxacin, gentamicin, and piperacillin/tazobactam) were administered appropriately <20% of the time.

Aim 2 is to calculate the frequency with which the appropriate cefazolin dose was administered, defined as 3 g of cefazolin for patients who weigh ≥120 kg versus 2 g of cefazolin for patients who weigh <120 kg. We hypothesized that underdosing (administration of 2 g instead of 3 g for patients ≥120 kg) occurred in more than 50% of eligible cases, indicating inconsistent adherence to weight-based dosing guidelines.

Aim 3 is to determine whether the likelihood of inappropriate administration differed based on the type of surgery. We hypothesized that adherence to antibiotic timing and dosing was lower in more complex surgical specialties, particularly orthopedics, due to longer preparation times and greater procedural variability.

## Materials and methods

Study design

This study was a secondary analysis of data originally recorded in the course of clinical care, i.e., a retrospective chart review designed to determine adherence with perioperative antibiotic administration. Ethical approval for this secondary analysis was obtained from the OhioHealth Institutional Review Board (no. 1824919-7). All data were de-identified prior to analysis to maintain patient confidentiality and compliance with the Health Insurance Portability and Accountability Act (HIPAA) regulations.

Participant information and identification

Potential study subjects were identified via a query of an existing OhioHealth database (e.g., CareConnect). The preliminary patient list generated from this query was reviewed and validated by the investigator or delegated study staff to ensure an accurate data pull and to confirm that all patients met the inclusion and exclusion criteria. The final study sample included patients who were 18 years or older, underwent outpatient surgery at OhioHealth Doctors Hospital between January 1, 2018, and December 31, 2021, and received antibiotics via injection, intravenous push (IVP), or intravenous piggyback (IVPB). Patients were excluded if they were inpatients, did not receive any antibiotics, or if the surgery time was not recorded. 

Number of participants

The maximum number of participants/records included for research was 2,573. This number was an estimate of the total number of cases that met the inclusion criteria, based on overall outpatient surgical volumes at Doctors Hospital. This sample size provided adequate statistical power, greater than 90% with an overall two-tailed alpha level of 5% to support the study hypothesis. If a higher-than-anticipated number of records met the inclusion criteria, a protocol amendment would have been submitted to increase the sample size. Of the 2,573 surgeries, we anticipated an unequal distribution across the antibiotics of interest, with approximately 80% of cases involving cefazolin (large therapeutic window), 15% involving clindamycin (narrow therapeutic window), and fewer than 10% involving vancomycin or other antibiotics (narrow therapeutic window). After applying inclusion and exclusion criteria and accounting for incomplete or missing records, 448 patient charts were ultimately analyzed. This reduction was due to incomplete antibiotic administration data and/or patient exclusion after validation.

Data collection

The data used for this study comprised information routinely collected and entered in the OhioHealth electronic medical record (Epic CareConnect) during the course of clinical care. This included demographics and patient characteristics (such as age, sex/gender, race/ethnicity, and medical comorbidities), diagnosis and diagnosis codes, surgical and procedure reports, and information about procedures or treatments received, including dates and times, locations, names of providers, and names of medications or procedures. The data were obtained through a query of OhioHealth electronic medical records and/or manual chart review.

Statistical analysis

All study data were summarized with descriptive statistics. Continuous variables were summarized with means, standard deviations, medians, and ranges. Categorical variables were summarized with counts and percentages. Aim 1 was assessed by using exact binomial 95% confidence intervals (CI) around the appropriate administration rate of antibiotics of interest. If the CIs did not contain the hypothesized rate, then we concluded that the data supported the claim. If the CIs did contain the hypothesized rate, then we concluded that there is not enough evidence to support the claim. For Aim 2, counts and percentages were reported for the subgroup of patients weighing more than or equal to 120 kg, comparing those who received the appropriate 3 g dose of cefazolin to those who received only 2 g. Aim 3 was assessed by comparing surgery types on inappropriate administration rates using chi-square tests or Fisher’s exact tests, as appropriate. No adjustments for multiple comparisons were made. Results with a p-value less than or equal to 0.05 were considered statistically significant.

## Results

To investigate adherence to perioperative antibiotic guidelines and its potential impact on surgical site infection risk, 448 participants met the inclusion criteria as listed in the “Participant Information and Identification” section. The average age was 54.6 years (SD = 15.7), with a median age of 56 years and a range from 18 to 88. 252 (56.3%) were female, and 348 (77.7%) identified as Caucasian. Moreover, 402 (89.7%) patients weighed under 120 kg, and 91 (20.3%) patients had diabetes. The most frequently performed surgical procedures were urology at 166 (37.1%), orthopedics with 114 (25.4%), and general surgery with 73 (16.3%) (Table 1).

**Table 1:** Demographics of the study participants N: number; SD: standard deviation

Parameter	Results
Age	-
N	448
Mean±SD	54.55 ± 15.70
Range	18.00 to 88.00
Median	56.00
-	-
Sex	-
Female	252 (56.3 %)
Male	196 (43.8 %)
-	-
Ethnicity	-
Not Hispanic or Latino	417 (93.1 %)
Hispanic or Latino	21 (4.7 %)
Declined to specify	10 (2.2 %)
-	-
Race	-
Caucasian	348 (77.7 %)
African American or Black	58 (12.9 %)
American Indian or Alaska Native	2 (0.4 %)
Asian	6 (1.3 %)
Native Hawaiian or Other Pacific Islander	1 (0.2 %)
Declined to Specify	31 (6.9 %)
Two or more races	2 (0.4 %)
-	-
Weight Category	-
<120 kg	402 (89.7 %)
≥120 kg	46 (10.3 %)
-	-
Diabetes	-
No	357 (79.7 %)
Yes	91 (20.3 %)
-	-
Surgery Category	-
Breast	7 (1.6 %)
General Surgery	73 (16.3 %)
Gynecology	33 (7.4 %)
Neurosurgery	22 (4.9 %)
Ophthalmology	1 (0.2 %)
Oral Surgery	1 (0.2 %)
Orthopedic	114 (25.4 %)
Otolaryngology	9 (2.0 %)
Pain management	1 (0.2 %)
Podiatry	6 (1.3 %)
Urology	166 (37.1 %)
Vascular	15 (3.3 %)

A total of 448 antibiotic administrations were analyzed. Cefazolin (ANCEF) IVPB 2 g was the most frequently administered antibiotic, accounting for 294 (65.6%) of all administrations. Other commonly administered antibiotics included clindamycin (Cleocin) IVPB 900 mg, administered in 64 (14.3%) cases, and ciprofloxacin 400 mg (Cipro) in 28 (6.3%) cases (Table 2). 

**Table 2:** Summary of antibiotic administration N: number; IVPB: intravenous piggyback; g: grams; mg: miligrams; NS: normal saline

Parameter	Results
N	448
Med Name	-
Cefazolin IVPB 2 g (premix)	294 (65.6%)
Clindamycin IVPB 900 mg (premix)	64 (14.3%)
Ciprofloxacin IVPB 400 mg (premix)	28 (6.3%)
Vancomycin 1500 mg in sodium chloride 0.9% (NS) 500 mL IVPB	27 (6.0%)
Cefotetan IVPB 2 g (premix)	10 (2.2%)
Cefazolin injection	2 (0.4%)
Vancomycin 1000 mg in sodium chloride 0.9% (NS) 200 mL IVPB	9 (2.0%)
Metronidazole IVPB 500 mg	8 (1.8%)
Gentamicin IVPB 80 mg (premix)	3 (0.7%)
Piperacillin-tazobactam IVPB 3.375 g (premix)	2 (0.4%)

Aim 1: antibiotic administration timing

Across all 448 patients, only 15 (3.3%) received antibiotics within the guideline-recommended time window before the incision (95% CI: 0.02-0.05). Conversely, 433 (96.7%) did not receive antibiotics within this timeframe (95% CI: 0.95-0.98). When analyzed by route of administration, IVP antibiotics were administered appropriately in 14 of 306 cases (4.6%) compared to one of 142 (0.7%) given by infusion pump. For IVP, 292 of 306 (94.5%) were administered outside the recommended window, while for the infusion-pump group, 141 of 142 (99.3%) were not given appropriately. This difference was found to be statistically significant (χ² = 3.37, p = 0.0446), and a Fisher’s exact test was used due to the small sample count.

Aim 2: appropriate cefazolin dosing by weight

For Aim 2, appropriate cefazolin dosing was evaluated based on patient weight. Among the 294 cases where cefazolin was administered, the dosing was found to be largely appropriate, as defined previously by the guidelines: 2 grams for patients weighing <120 kg and 3 grams for those ≥120 kg. A total of 261 (88.8%) cases received appropriate cefazolin dosing, with a 95% CI of 0.85-0.92. Conversely, 33 (11.2%) cases received inappropriate dosing, with a 95% CI of 0.08-0.15.

Aim 3: timing of antibiotic administration by surgery type

Aim 3 looked into the likelihood of inappropriate antibiotic administration based on the type of surgical procedure, both for individual procedures and predefined groups (Tables 6, 7). Appropriate administration rates varied across individual surgery types, although most remained low. Several surgery categories demonstrated 0.0% appropriate administration, such as breast surgeries, general surgery, vascular, and others. Among categories with some appropriate administration, orthopedic procedures had the highest rate with 9/114 (7.9%) cases, followed by gynecology at 2/33 (6.1%) cases, neurosurgery at 1/22 (4.5%) cases, and urology at 3/166 (1.8%) cases (Table 3).

**Table 3:** Appropriate timing of antibiotic administration by surgical type

Surgery	Appropriate administration
Breast	0 / 7 (0.0%)
General surgery	0 / 73 (0.0%)
Gynecology	2 / 33 (6.1 %)
Neurosurgery	1 / 22 (4.5 %)
Ophthalmology	0 / 1 (0.0%)
Oral Surgery	0 / 1 (0.0%)
Orthopedic	9 / 114 (7.9 %)
Otolaryngology	0 / 9 (0.0%)
Pain management	0 / 1 (0.0%)
Podiatry	0 / 6 (0.0%)
Urology	3 / 166 (1.8 %)
Vascular	0 / 15 (0.0%)

To further analyze potential differences and account for variations in case volume, surgery types were categorized into four predefined groups, as seen in Table 4. Group A included general surgery, urology, breast, and gynecological procedures, with an appropriate antibiotic timing rate of 5/279 (1.79%) cases. Group B, which consisted of neurosurgery and pain management cases, had a slightly higher compliance rate at 1/23 (4.35%). Group C, which included oral surgery, ophthalmology, and otolaryngology, had no cases of appropriately timed antibiotic administration. Group D, made up of orthopedic, podiatry, and vascular surgeries, had the highest compliance among the groups, with 9/135 (6.67%). A statistical comparison of appropriate administration rates across these four broad surgical groupings yielded a p-value of 0.068. This value, being greater than the conventional significance level of 0.05, indicated that there was no statistically significant difference in appropriate administration rates among these grouped surgical categories.

**Table 4:** Antibiotic timing by surgical group Chi-squared test was used to calculate the p-value. Group A: General Surgery Group (general surgery, urology, breast, and gynecological procedures). Group B: Neurosurgery Group (neurosurgery and pain management procedures). Group C: Facial Surgery Group (oral surgery, ophthalmology, otolaryngology). Group D: Orthopedic-Vascular Group (orthopedic, podiatry, vascular) n = number

Surgery	Group A (n = 279)	Group B (n = 23)	Group C (n = 11)	Group D (n = 135)	X^2^ value	p-value
Appropriate administration, n(%)	5 (1.79)	1 (4.35)	0 (0.00)	9 (6.67)	7.13	.068

## Discussion

This analysis of perioperative antibiotic use across a large and diverse surgical population highlighted an ongoing gap between clinical guidelines and real-world practice. Cefazolin was typically dosed appropriately; however, the timing of administration, which is possibly the most important factor in preventing infections, was remarkably subpar. There are serious concerns regarding patient safety and protocol adherence, considering a large number of patients did not receive antibiotics within the recommended window preceding incision [16,17].

These results supported the existing literature's findings that noncompliance with perioperative antibiotic scheduling is an ongoing issue in various specialties and institutions [18,19]. Timely antibiotic administration is a cornerstone of surgical site infection prevention, and even small deviations from the recommended pre-incision window may decrease the antibiotic's protective effect [20]. Despite this known knowledge, our findings implied that timing approaches are either not adequately included in preoperative workflows or are applied inconsistently [21]. Improving adherence to this timing, potentially through more standardized protocols and surgical safety checklists, remains a key target for quality improvement in perioperative care [19]. 

One significant finding from the data was that, in contrast to antibiotics given via infusion pumps, those given via IV push showed noticeably better compliance with scheduling standards. This variation may be a consequence of operational inefficiencies; infusion pump preparations take much longer and may be delayed because of staff workloads or lack of communication in the operating room. By contrast, IV push has been shown to minimize delays related to pharmacy preparation, nursing workflow, and equipment setup, all of which are common contributors to poor timing in the operating room. With this being said, simplifying the antibiotic delivery through IV push methods can increase compliance and decrease timing errors [22].

An additional sign of a systems-level concern was the variation in adherence between surgical specialties. For instance, slightly higher compliance was seen in orthopedic cases, which may be due to tighter perioperative protocols or more frequent checklist use. Nearly zero compliance was seen in other surgical specialties, such as urology and general surgery, which may indicate differences in preoperative planning or a lack of established protocols. It has been shown that employing specialty-specific preoperative checklists, especially in conjunction with anesthesia timeouts, enhances antibiotic scheduling adherence and lowers the risk of infection [23].

Although cefazolin dosing by weight was generally appropriate, the proportion of patients who received wrong doses, specifically those weighing more than or equal to 120 kg, highlighted another area of attention. Studies have consistently shown that obese patients are more likely to receive subtherapeutic prophylaxis due to unclear dosing procedures or a failure to alter normal orders, while having a higher risk of surgical infections [24,25]. It would be useful and effective to modify electronic order sets to automatically indicate the right dosages and recognize patients who weigh more than a certain amount to reduce the number of patients receiving incorrect doses of cefazolin [26].

The results of this study also have broader practical ramifications. Higher rates of infection, longer stays in the hospital, and higher expenses are all consequences of improper prophylactic timing that have a direct impact on quality metrics and institutional performance. Hospitals that aim to improve surgical outcomes and reduce postoperative complications may need to consider incorporating antibiotic timing guidelines directly into the electronic health record (EHR) workflow, necessitating an anesthesiologist or nurse confirmation prior to incision. Systems with audit-feedback features and immediate alerts have shown promising results in terms of reducing infection rates and increasing compliance with the guidelines [27].

While the study benefited from a large sample size and a cross-specialty approach, it was inherently limited by its retrospective design and the absence of clinical outcome data such as infection rates. Therefore, even while the research sufficiently indicated a solid relationship, the study was unable to completely ascertain the precise effects of timing errors. Furthermore, noncompliance causes that should be further investigated qualitatively, like postponed case commencement, ambiguous role assignments, or electronic medical record challenges, were not captured. The reliance on documentation accuracy and manual data validation also presented potential sources of information bias.

## Conclusions

Perioperative antibiotic prophylaxis remains a critical component of SSI prevention, yet there is a persistent gap between clinical guidelines and real-world practice. More than just clinical understanding is required to enhance perioperative antibiotic scheduling; operational discipline, standard processes, and real-time accountability are also required. Automated dosing prompts, EHR-integrated checklists, quicker administration routes, and interdisciplinary interactions are just a few of the system-level improvements that should result from our findings, which represent a larger national problem. Future research should examine the impact of these interventions on surgical outcomes and compliance across different clinical contexts.
